# False Positive or False Negative—An Interesting Case in Prenatal Diagnostic Laboratory

**DOI:** 10.1002/jcla.70145

**Published:** 2025-12-12

**Authors:** Pingping Zhang, Yanmei Sun, Haishen Tian, Xuedong Shi, Limin Rong, Yali Li

**Affiliations:** ^1^ Department of Reproductive and Genetics Hebei General Hospital Shijiazhuang Hebei China

**Keywords:** BACs‐on‐Beads, fluorescence in situ hybridization, karyotype analysis, prenatal diagnosis, SNP‐Array analysis

## Abstract

**Background:**

Prenatal diagnosis relies on diverse clinical laboratory techniques to assess the health status of a developing fetus in utero. When multiple diagnostic methods applied to the same fetal sample yield discordant results, some suggestive of abnormalities and others normal, this discrepancy poses substantial challenges to accurate clinical interpretation and decision‐making.

**Case Presentation:**

A 23‐year‐old pregnant woman underwent non‐invasive prenatal testing (NIPT) that yielded an abnormal fetal screening result. Subsequent prenatal diagnostic assessments, including single nucleotide polymorphism (SNP)‐Array analysis and prenatal BACs‐on‐Beads (PNBoBs) assay, suggested a normal fetal karyotype. However, conventional karyotypic analysis and fluorescence in situ hybridization (FISH) uncovered fetal chromosomal abnormalities.

**Conclusions:**

Different diagnostic techniques possess distinct strengths, limitations, and applicable scopes. Certain abnormalities may evade detection by a single technique due to technical constraints or sample‐specific biological characteristics. Therefore, in prenatal diagnosis, clinicians should select suitable diagnostic modalities based on clinical context. When necessary, multiple complementary methods should be employed for cross‐validation to approximate the true fetal status and avoid missed diagnoses and misdiagnoses.

## Graphical abstract

A prenatal case in which discordant results were identified among SNP‐Array, PNBoBs, conventional karyotyping, and FISH, highlighting the importance of recognizing the limitations of various testing techniques for clinicians to avoid misdiagnosis and missed diagnosis.

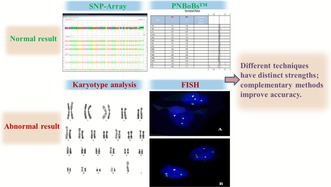

## 1 Introduction

Prenatal diagnosis, also termed intrauterine diagnosis, refers to a technical approach designed to evaluate whether a fetus is affected by specific genetic disorders or congenital abnormalities prior to birth. With informed consent obtained, biological specimens including umbilical cord blood, amniotic fluid, or chorionic villi are collected and analyzed using advanced cytogenetic or molecular genetic technologies—procedures that constitute a core component of the prenatal diagnostic workflow. Currently, the primary laboratory techniques employed in prenatal diagnosis include karyotyping, single nucleotide polymorphism (SNP) array analysis, prenatal BACs‐on‐Beads (PNBoBs) testing, fluorescence in situ hybridization (FISH), and various sequencing technologies. A key challenge lies in preventing children with congenital defects from imposing emotional and psychological burdens on their families, while concurrently mitigating the risk of unnecessary termination of pregnancies involving potentially healthy fetuses. To address this, prenatal diagnostic laboratories typically adopt at least two complementary testing modalities to ensure diagnostic accuracy and reliability. Integrating results from multiple diagnostic approaches enables expectant parents to make more informed reproductive choices when confronted with the difficult decision of continuing or terminating a pregnancy. While the majority of pregnant women aspire to have a healthy, developmentally normal fetus, prenatal diagnostic procedures inevitably elicit anxiety, particularly when results from different testing modalities are inconsistent or conflicting. Appropriate genetic counseling can significantly alleviate maternal anxiety and reduce the risk of medical disputes. This article presents a clinical case involving discordant prenatal diagnostic results, aiming to provide references and guidance for clinical practice in similar scenarios.

## 2 Case Presentation

### 2.1 Clinical Features

A 23‐year‐old primigravida (gravida 1, para 0) underwent non‐invasive prenatal testing (NIPT) at 17 weeks of gestation at Jizhou Maternal and Child Health Care Hospital. The NIPT assay was performed as previously described by Li et al. [1], yielding negative results for trisomy 21, trisomy 18, and trisomy 13, but positive for sex chromosome aneuploidy, specifically, a numerical reduction in sex chromosome copy number compared to the normal karyotype. The patient was subsequently referred to our hospital at 21 weeks of gestation for further prenatal diagnosis and genetic counseling. The couple had no consanguineous relationship and were both phenotypically healthy, with unremarkable personal medical histories and family backgrounds. Fetal ultrasonography revealed focal echogenic enhancement in the lower abdominal region. Following detailed counseling regarding potential risks, the patient opted for invasive prenatal diagnosis. Amniocentesis was performed under real‐time ultrasound guidance to collect amniotic fluid samples, which were subsequently subjected to prenatal karyotyping and single nucleotide polymorphism (SNP)‐Array analysis.

### 2.2 Giemsa (G)‐Banding Karyotype Analysis

Conventional G‐banding karyotyping was performed on cultured amniotic fluid cells at a resolution of approximately 320 bands. A total of 30 mL amniotic fluid was collected and dispensed into two sterile capped centrifuge tubes under aseptic conditions, followed by centrifugation at 1500 rpm for 8 min. The two tubes were simultaneously cultured in double lines using media from two manufacturers (Guangzhou Baiyunshan Biopharmaceutical Co. Ltd. and Guangzhou Heneng Biotechnology Co. Ltd.). The supernatant was discarded, and the pellet was resuspended in 4 mL of amniotic fluid cell culture medium per tube. Each resuspended sample was inoculated into two separate cell culture flasks, which were then incubated in a 5% CO_2_ incubator at 37°C with regular medium changes. The culture period typically ranged from 9 to 15 days before harvest. Fresh culture medium was replaced 24 h prior to harvest to ensure adequate cell nutrition. Colchicine was added to each flask at a final concentration of 0.05 μg/mL 4–6 h before the end of incubation. For cell harvesting, the culture medium was aspirated, and 4 mL of EDTA–trypsin digestive solution was added to each flask, which was then incubated at 37°C for 5 min. The cells were rinsed 3–4 times with a pipette by aspiration, and the cell suspension was transferred to centrifuge tubes. After centrifugation at 2000 rpm for 10 min, the supernatant was discarded, leaving 0.2–0.3 mL of cell pellet. Subsequently, 6 mL of preheated hypotonic solution (1% sodium citrate and 0.075 M KCl at a 1:1 ratio) was added, and the mixture was gently pipetted to homogenize, followed by hypotonic treatment at 37°C for 25 min. A total of 1 mL of fixative (methanol:glacial acetic acid = 3:1) was added dropwise with gentle bubbling to initiate fixation. After centrifugation at 2000 rpm for 10 min, the supernatant was discarded, and 6 mL of fresh fixative was added for 40 min of primary fixation. Following another round of centrifugation, the supernatant was removed, and 4 mL of fixative was added for a second fixation step (20 min) with gentle bubbling. After the final centrifugation at 2000 rpm for 10 min, the supernatant was discarded, and 3–4 drops of fresh fixative were added to resuspend the pellet with bubbling. The cell suspension was then dropped onto clean glass slides for chromosome spreading. The prepared slides were baked in an 80°C oven for 3 h prior to G‐banding.

**FIGURE 1:**
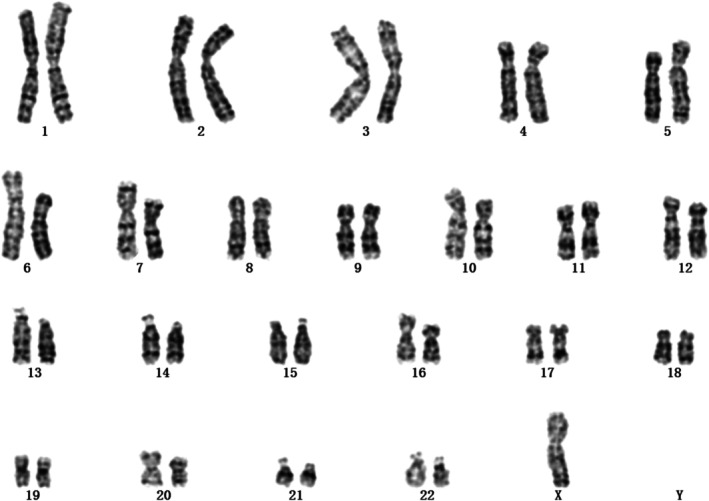
The first G‐band karyotype analysis of the foetus (20 metaphases were counted and 5 mitotic figures were analyzed) indicated a result of 45,X.

G‐banding karyotypic analysis was performed in accordance with the International System for Human Cytogenomic Nomenclature (ISCN) 2016. For the amniotic fluid sample, a total of 20 metaphases were counted, with 5 mitotic spreads subjected to detailed analysis. Initial cytogenetic assessment identified an abnormal karyotype of 45,X (Figure 1), which was consistent with the prior NIPT findings.

### 2.3 SNP‐Array Analysis

SNP array analysis was performed using the Affymetrix CytoScan 750K SNP array (Affymetrix, Santa Clara, CA, USA) on genomic DNA extracted from pelleted cells of 10 mL uncultured amniotic fluid. Genomic DNA was subjected to digestion, ligation, PCR amplification, purification, fragmentation, and labeling, followed by hybridization to the Affymetrix 750K array, which contains 200,000 SNP markers and 550,000 CNV markers. Following array washing, staining, and scanning, raw data were analyzed using Chromosome Analysis Suite software (version 4.0.0.385; R28959; Thermo Fisher Scientific Inc.).

**FIGURE 2:**
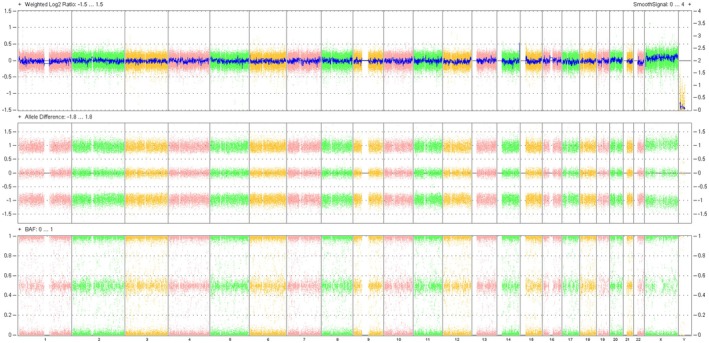
SNP‐array analysis showed that the foetus has a normal result of arr(X,1–22) × 2.

**FIGURE 3:**
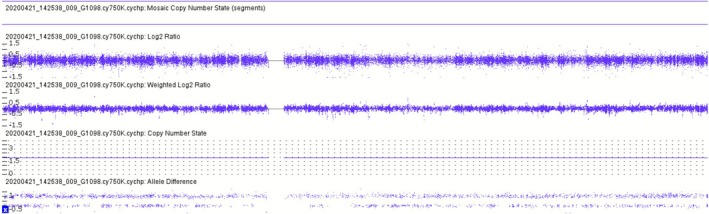
The detail view report of chromosome X revealed by SNP‐array.

The SNP‐array analysis revealed a normal result: arr(X,1–22) × 2 (Figure 2), with a detailed view of chromosome X provided in Figure 3. This finding was entirely discordant with the abnormal karyotypic result, despite the latter being consistent with the prior NIPT outcome. Could this discrepancy indicate a false negative in the SNP‐array assay?

### 2.4 Prenatal BACs‐on‐Beads (PNBoBs) Assay

Prenatal BACs‐on‐Beads (PNBoBs) assay was performed using the BoBs platform (PerkinElmer Wallac, Turku, Finland) on genomic DNA extracted from pelleted cells of 8 mL uncultured amniotic fluid. Genomic DNA was labeled, purified, and hybridized for 16–18 h in accordance with the manufacturer's protocol. Following washing and staining, fluorescence signal intensity was detected using the Luminex200 system. Raw data were analyzed with BoBsoft 2.0 software (PerkinElmer Wallac, Turku, Finland) utilizing the Male/Female Reference DNA algorithm.

**FIGURE 4:**
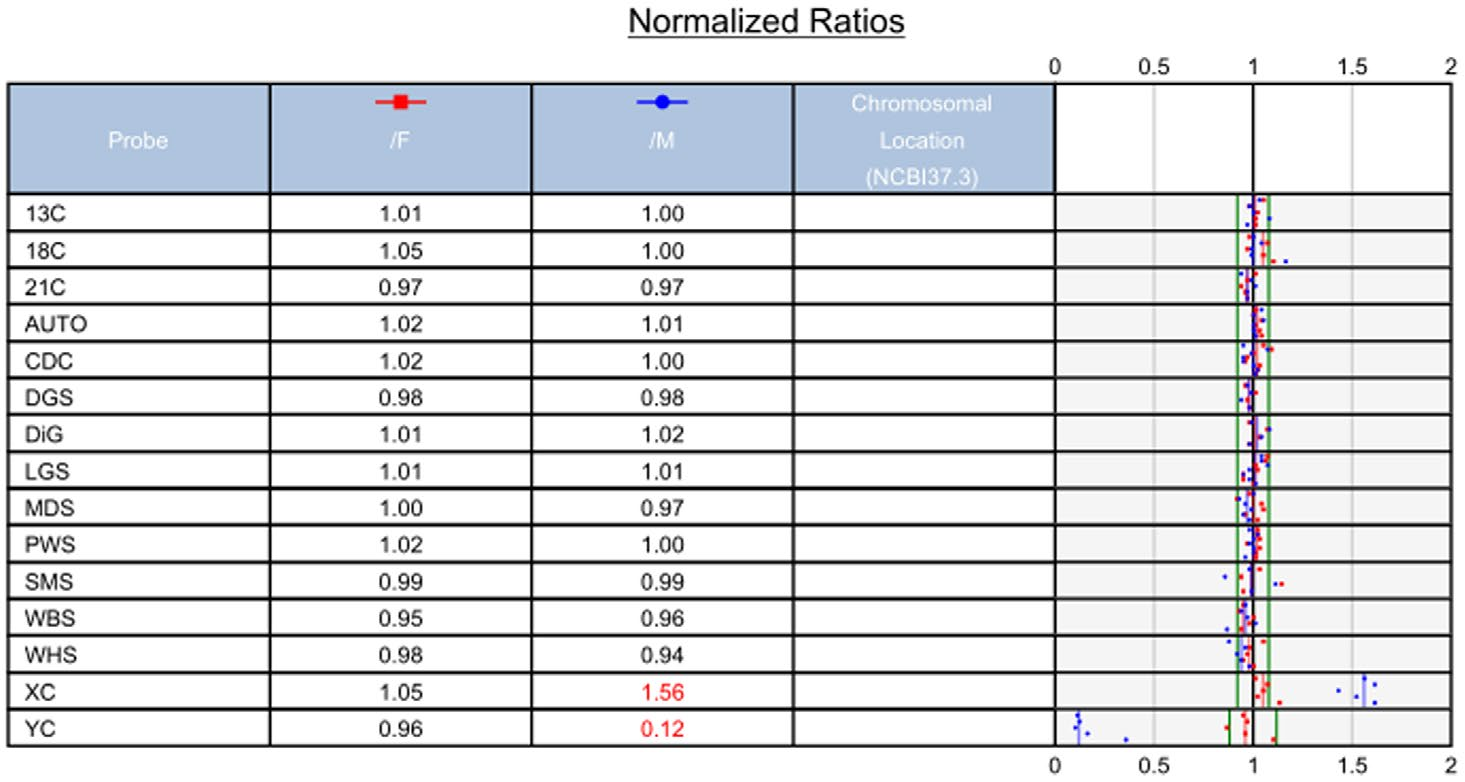
The PNBoBs analysis revealed a normal result of 46,XX.

The PNBoBs assay yielded a completely normal result of 46,XX (Figure 4), consistent with the SNP‐array findings. Could the prior G‐banding karyotypic analysis have yielded a false positive?

### 2.5 Fluorescence In Situ Hybridization (FISH) Analysis

FISH analysis was performed targeting chromosomes 18, X, and Y using specific probes (Beijing GP Medical Technology Co. Ltd., Beijing, China) in accordance with the manufacturer's instructions. The probes targeted centromeric regions: CSP18 (18p11.1‐q11.1), CSPX (Xp11.1‐q11.1), and CSPY (Yp11.1‐q11.1). The centromere of chromosome X was labeled with spectrum green, chromosome 18 with spectrum sky blue, and the Y chromosome centromere (labeled with spectrum red) was not detected in the specimen.

**FIGURE 5:**
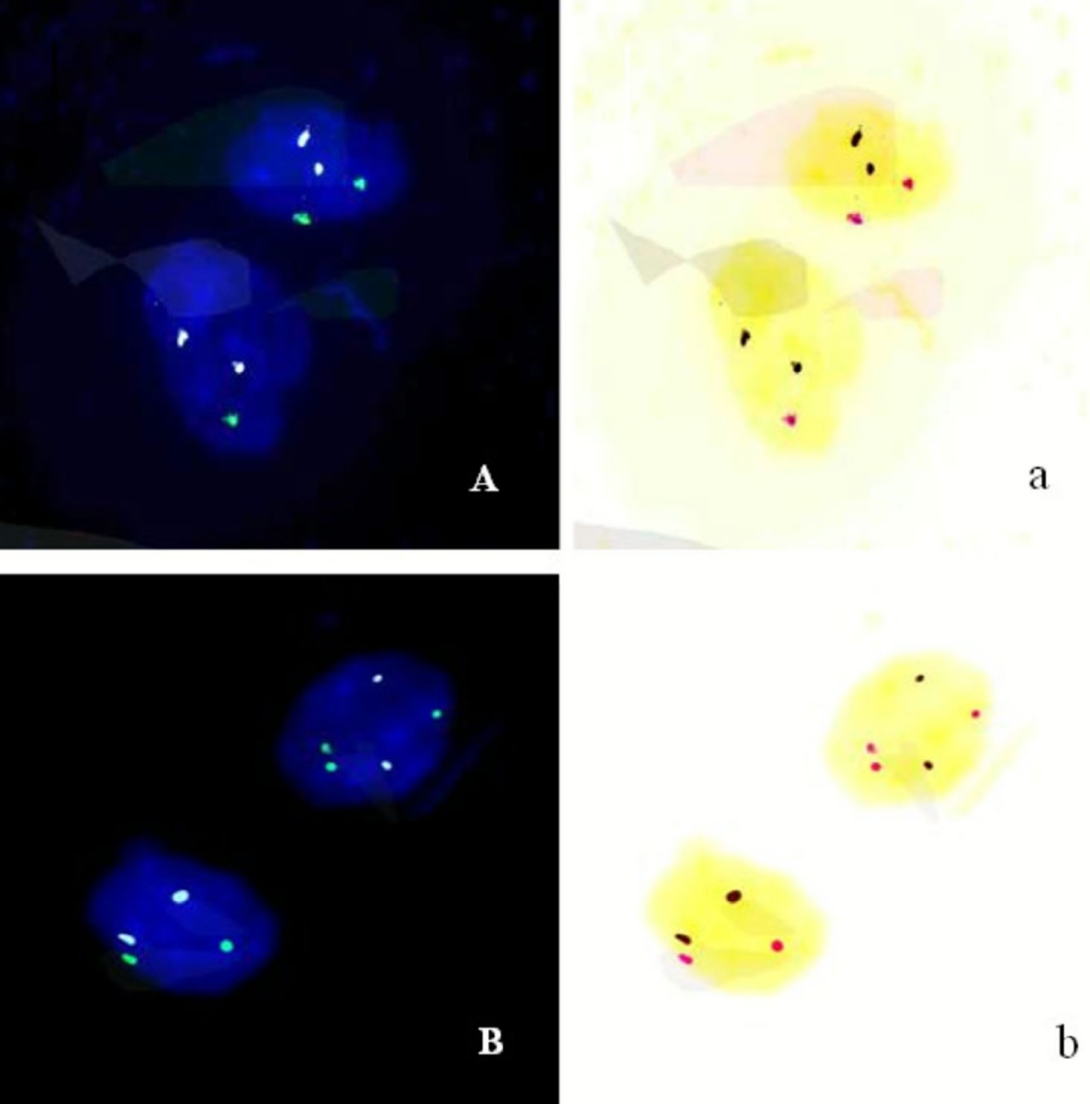
FISH analysis revealed a result of Mos 46,XX[62]/45,X[24]/47,XXX[14]. (A) The FISH results of 46,XX and 45,X. Green represents X chromosome centromere signal points; Cyan represents centromere signal points on chromosome 18. Panel (a) is the reverse color image of (A). In the reverse color image red is represents the centromere signal points on the X chromosome. (B) The FISH results of 46,XX and 47,XXX. Green represents X chromosome centromere signal points; cyan represents centromere signal points on chromosome 18. Panel (b) is the reverse color image of (B). In the reverse color image red represents the centromere signal points on the X chromosome.

Cells were randomly selected for counting if they met the following criteria: clear and distinguishable signals across all channels, uniform signal intensity, smooth signal edges, a clean background, and no signal overlap. Areas with uneven hybridization, cells with indistinct nuclear contours or overlapping nuclei, and regions with excessively dark backgrounds that hindered accurate signal judgment were excluded from analysis. Typically, 50 cells were randomly counted per probe set. Fifty uncultured amniocytes from the sample were analyzed using a Nikon Eclipse 80i fluorescence microscope. If chromosomal aberrations were detected, the count was expanded to 100 amniocytes for further analysis. In the event of chromosomal mosaicism, the cell count was extended to 100–500 cells to accurately determine the mosaicism ratio. Finally, a FISH result of nuc ish Mos 46,XX[62]/45,X[24]/47,XXX[14] was obtained (Figure 5).

## 3 Discussion

Karyotype analysis has long been regarded as the “gold standard” for diagnosing chromosomal abnormalities [2], as it provides a comprehensive overview of the entire chromosomal complement and affords unique advantages for detecting translocations. However, it has a relatively low resolution (approximately 5–10 Mb) [3], which restricts its capacity to identify microdeletions, microduplications, and loss of heterozygosity (LOH). In contrast, SNP‐Array technology—with significantly higher resolution—can detect LOH, microdeletions, and microduplications greater than 100 kb. Nevertheless, it cannot identify balanced translocations in the absence of prior cell culture [4]. Combining karyotype analysis with SNP‐Array enables complementary verification and improves diagnostic accuracy, rendering it one of the most widely used method combinations in prenatal diagnosis [2]. Prenatal PNBoBs testing not only detects common aneuploidies (e.g., trisomies 13, 18, and 21) and sex chromosome abnormalities (X and Y) but also concurrently identifies nine high‐prevalence microdeletion syndromes. Its primary advantage is the elimination of the need for cell culture, facilitating rapid diagnosis [5]. FISH utilizes fluorescently labeled oligonucleotide probes that hybridize to target DNA sequences in the sample, with results obtained by detecting fluorescence signals using a fluorescence microscope. By directly analyzing uncultured interphase cells, FISH provides rapid, highly specific, and visually intuitive results, making it the preferred method for mosaicism detection [6, 7].

**FIGURE 6:**
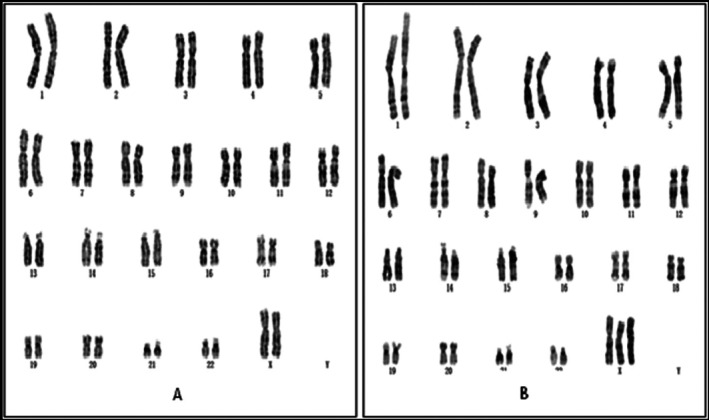
The second G‐band karyotype analysis of the foetus:In addition to the previously discovered 45,X, two other karyotypes 46,XX(A) and 47,XXX(B) were found. A result of Mos 45,X[43]/47,XXX[7]/46,XX[5] was finally exposed.

In this case, multiple distinct experimental techniques were applied to analyze the same prenatal diagnostic sample, resulting in inconsistent findings. Karyotypic analysis identified an abnormal karyotype (Figure 1), which was consistent with the previously observed sex chromosome hypoploidy detected by NIPT. In contrast, SNP‐Array and prenatal PNBoBs assays indicated a normal female fetus. Fluorescence in situ hybridization (FISH) further revealed that the fetus harbored sex chromosome mosaicism: 46,XX[62]/45,X[24]/47,XXX[14]. These findings are both seemingly contradictory and mutually complementary, and cannot be simply categorized as false‐positive or false‐negative results. Each detection method possesses unique technical advantages, limitations, and applicable scopes. Due to the inherent characteristics of the sample, certain conditions may fall outside the detection range of specific technologies, leading to missed detections or “escape” phenomena. NIPT is a screening tool rather than a diagnostic modality, and carries an inherent risk of false‐positive results—particularly in the assessment of sex chromosome abnormalities [8]. Karyotypic analysis requires cell culture, during which differential growth advantages of distinct cell lines may alter the observed mosaic proportions. In this case, the 45,X cell line exhibited greater growth dominance during culture, while the 46,XX and 47,XXX cell lines proliferated less efficiently. Consequently, only the dominant 45,X cell line was detected in the initial karyotypic analysis, which failed to accurately reflect the true mosaic ratio. Guided by the FISH results, a second karyotypic analysis was performed with an expanded number of metaphases counted, ultimately yielding the mosaic karyotype: Mos 45,X[43]/47,XXX[7]/46,XX[5] (Figure 6).

Therefore, karyotypic analysis should be supplemented with uncultured cell‐based assays to enable more comprehensive result interpretation. Both SNP‐Array and prenatal PNBoBs assays involve extracting genomic DNA from a large number of cells—including all cell lineages present in the sample—to evaluate whether the X chromosome dosage falls within the normal range. In this specific case, two abnormal chromosomal lineages (45,X and 47,XXX) were identified: one with reduced X chromosome copy number and the other with increased copy number. These two lineages were present in roughly equivalent proportions, and their opposing effects on the overall X chromosome dosage offset each other, leading to a false impression of normal X chromosome dosage in the SNP‐Array and PNBoBs results. FISH serves as a confirmatory diagnostic tool when results from other genetic testing modalities are inconclusive. In the present case, FISH analysis confirmed three distinct cell lines: 45,X, 46,XX, and 47,XXX, resolving the discrepancies between the initial karyotypic, SNP‐Array, and PNBoBs findings.

Turner syndrome (TS) is caused by the partial or complete absence of a second sex chromosome in some or all cells. Mosaic TS involving trisomy X (45,X/47,XXX or 45,X/46,XX/47,XXX) is rare, accounting for approximately 3% of all TS cases [9]. The core clinical manifestations of TS include gonadal dysgenesis and short stature, with additional features such as distinct cardiovascular anomalies, characteristic facial phenotypes, renal abnormalities, and autoimmune thyroid disorders. Mosaic TS exhibits a broad phenotypic spectrum, which is influenced by multiple factors including the mosaicism subtype, tissue distribution of abnormal cell lines, and the proportion of aberrant cells. Currently, there is a paucity of reliable references for the prognostic assessment of mosaic TS fetuses—particularly those with multiple cell line mosaicism. Chen et al. reported a case where initial amniocentesis identified a 45,X/46,XX karyotype, while repeat amniocentesis revealed 45,X[17]/47,XXX[8]/46,XX[121]; the pregnancy ultimately resulted in a favorable fetal outcome at birth [10]. However, long‐term follow‐up data during adolescence were not available for this patient.

Following comprehensive prenatal diagnosis and genetic counseling, the couple opted to terminate the pregnancy.

In conclusion, clinicians engaged in prenatal diagnosis must have a thorough understanding of the strengths and limitations of various genetic testing modalities. They should select suitable techniques based on clinical scenarios and, when necessary, employ multiple complementary modalities for cross‐validation. This approach not only improves diagnostic accuracy but also effectively reduces the risks of misdiagnosis and missed diagnosis in clinical practice.

## Author Contributions

Yanmei Sun collected the patient clinical information. Haishen Tian, Limin Rong, and Xuedong Shi performed the experiments. Pingping Zhang analyzed the data. Pingping Zhang and Yali Li drew the manuscript. All authors read and approved the final manuscript.

## Funding

This study was supported by the Medical Technology Tracking and Application Project of Hebei Province (GZ2024008), the Science and Technology Support Plan of Hebei Province (22377792D), the High‐Level Talents Funding Project of Hebei Province (C20231018), and the Government‐Funded Project on Clinical Medical Talent Training (ZF2023191).

## Conflicts of Interest

The authors declare no conflicts of interest.

## Data Availability

Data will be made available on request.
